# The Clinical Effects of Steroids Therapy in the Preserving Residual Hearing after Cochlear Implantation with the OTICON Neuro Zti EVO

**DOI:** 10.3390/jcm10132868

**Published:** 2021-06-28

**Authors:** Magdalena B. Skarzynska, Aleksandra Kolodziejak, Elżbieta Gos, Piotr H. Skarzynski

**Affiliations:** 1 Institute of Sensory Organs, 05-830 Warsaw, Poland; p.skarzynski@csim.pl; 2 Center of Hearing and Speech Medincus, 05-830 Warsaw, Poland; 3 Department of Teleaudiology and Screening, World Hearing Center, Institute of Physiology and Pathology of Hearing, 05-830 Warsaw, Poland; a.kolodziejak@ifps.org.pl (A.K.); e.gos@ifps.org.pl (E.G.); 4 Heart Failure and Cardiac Rehabilitation Department, Faculty of Medicine, Medical University of Warsaw, 03-242 Warsaw, Poland

**Keywords:** partial deafness treatment, steroids, cochlear implants

## Abstract

Background: A prospective clinical study was conducted to investigate whether two different pharmacotherapy strategies of steroid administration impact hearing preservation in adult patients who underwent cochlear implantation with the Oticon Medical Neuro cochlear implant system. Methods: Twenty nine adult participants were included. Pure tone audiometry was performed before implantation, during processor activation and 12 months after activation. There were three treatment groups: (1) intravenous steroid therapy (standard steroid therapy with dexamethasone administrated intravenously at the dose 0.1 mg/kg body mass twice a day); (2) combined oral and intravenous steroid therapy (extended steroid therapy with dexamethasone administrated intravenously at the dose 0.1 mg/kg b.m. twice a day and prednisone (orally) at the dose 1 mg/kg body mass/24 h), and (3) no steroid therapy (a control group). Patients’ hearing thresholds before implantation were on average 103 dB HL, 89 dB HL, and 93 dB HL, respectively. Results: Deterioration of hearing thresholds was observed in all three patients’ groups. Twelve months after surgery the patients with and without steroid therapy had similar hearing thresholds. Conclusions: The steroid regimen used in this study did not play a significant role in patients with non-functional residual hearing, who underwent cochlear implantation with the Oticon Medical Neuro cochlear implant system.

## 1. Introduction

Cochlear implantation (CI) is widely regarded as one of the most effective treatments for patients with severe to profound hearing loss [1,2,3,4,5]. It is estimated that there are over 220,000 implant users worldwide today. In the United States, as of December 2010, approximately 43,000 adults and 28,000 children have had the device implanted [6]. Initially, it was assumed that hearing implant surgery would result in complete loss of residual hearing, but it turned out that preserving hearing in CI surgery is possible [7]. The treatment of partial and profound deafness has been a great clinical challenge. In particular, this applies to patients whose hearing in the diagnostic ranges and quality was normal. and the only criteria were its electrical complement in the medium and high frequency range. The pioneering implantation of CI in an adult with partial deafness, carried out in 2002 by Professor H. Skarzynski, and in 2004 in a child with only such hearing damage has set a new direction for research into various partial, increasingly common hearing losses [8]. To optimize sound perception, preservation of residual hearing is a secondary goal of implantation [9,10]. A potentially soft approach could be achieved also with an endometal approach, but in our experience this should be reserved for patients, with a cutting-edge, limited approach, via mastoid [11,12]. In the last few years, attention has been paid to the use of glucocorticoids as a potential pharmacological intervention to protect residual hearing, which is especially important for the perception of speech, music, sound localization and hearing during noise [13,14]. The technique reduces inflammation, which is one of the potential causes of cochlea damage in cases where electrode insertion has been relatively atraumatic [15]. Dexamethasone is a synthetic glucocorticoid that has immunosuppressive and anti-inflammatory properties. It is used in the treatment of diseases such as asthma, arthritis, adrenal insufficiency or chronic obstructive pulmonary disease. In the field of otorhinolaryngology, dexamethasone and other glucocorticoids (e.g., prednisone, methylprednisolone) are used in the treatment of severe and chronic diseases, such as partial deafness, Meniere’s disease or autoimmune diseases [16,17].

### 1.1. Milestones in the History of the Oticon Neuro Zti

Oticon Medical’s Neuro cochlear implant (CI) system consists of the Neuro Zti implant and the Neuro sound processor (first generation: Neuro 1; second generation: Neuro 2). This implant has a titanium base and a zirconia casing (hence the designation Zti) and has been available since 2015. It is compatible with two types of 20-channel electrode array, the Classic and the EVO. The Classic electrode array, stiffer than the EVO electrode array, is better suited for challenging anatomical situations. The EVO electrode array is better suited for a soft surgery approach [18]. An EVO electrode was used in our patients. The carrier of the EVO electrodes was created to protect the delicate structure of the cochlea, which is especially important for preserving residual hearing. This electrode has a smooth surface, small dimensions, a thin tip and is flexible. It has been designed to ensure that non-traumatic introduction and as a result maximum cochlea protection met the necessary conditions [19].

### 1.2. Aim of the Study

The main aim of this study was to assess two different regimes of steroids therapy (dexamethasone versus dexamethasone plus prednisone) for hearing preservation (residual hearing) in adult patients who underwent cochlear implantation with the Oticon Medical Neuro cochlear implant system (Neuro Zti implant, Neuro 2 sound processor and Zti EVO atraumatic electrode). Enrolled patients were randomly assigned to one of three subgroups: (1) intravenous only steroid therapy (dexamethasone, 0.1 mg/kg body mass (body weight) /12 h, (2) combined steroid therapy (dexamethasone, 0.1 mg/kg body mass (body weigh) administrated twice a day plus prednisone at a dose of 1 mg/kg body mass/24 h with a reduction of dose over time) and (3) a control group (no steroid therapy). This research is a continuation of previous clinical research with the aim of optimizing pharmacological steroid treatment in different cochlear implantation systems. 

## 2. Materials and Methods

### 2.1. Ethical Approval and Patients Studied

**Figure 1:**
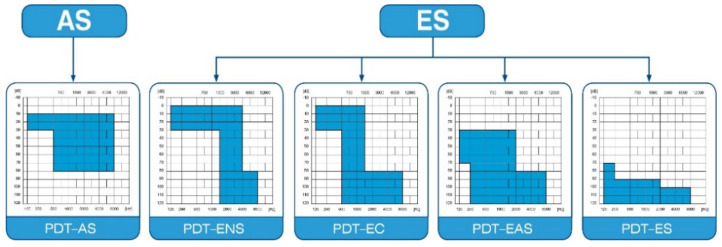
Partial deafness treatment groups for cochlear implantation. AS—acoustic stimulation; ENS—electro–natural stimulation; EC—electrical complement; EAS—electrical–acoustic stimulation; ES—electrical stimulation.

The protocol of this prospective clinical trial was approved by the Bioethics Committee of the Institute of Physiology and Pathology of Hearing in Kajetany (IFPS: KB/06/2016) and was in line with the Declaration of Helsinki. Patients enrolled in the study suffered from severe or profound hearing loss, and were classified according to Skarzyński’s classification of partial deafness treatment (PDT) (Figure 1): having PDT EAS—acoustic enhancement of preserved hearing residues—or PDT ES—electrical stimulation of existing non-functional hearing residues [10].

To be included in the study the eligibility criteria were as follows: Adult patients who qualified for cochlear implantation with hearing loss in the range 65–120 dB at frequencies 0.25–1 kHz and from 75 dB–120 dB at 2–8 kHz. The criteria for exclusion from the study were as follows: comorbidity: hypertension, diabetes or cancer, and contraindications for steroid therapy (taking drugs that may weaken or intensify the effects of steroids: anticoagulants, antidepressants, immunosuppressants), and hypersensitivity to the drug or its components. The inclusion criteria are in line with the international HEARRING Group consensus on hearing protection for cochlear implant users [20].

### 2.2. Therapeutic Patients Groups

**Figure 2:**
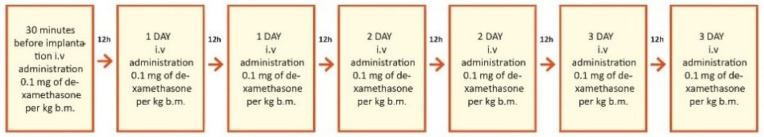
Study design for the first group, treated with intravenous steroid only. i.v.—intravenous; b.m.—body mass = body weight.

**Figure 3:**
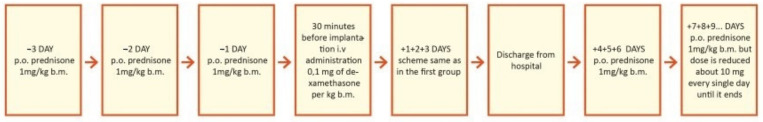
Study design for second group, treated with combined oral and intravenous (iv) steroid therapy (prolonged therapy). p.o.—per oral; i.v.—intravenous; b.m.—body mass = body weight.

This study was prospective and included 29 patients who planned to undergo cochlear implantation. They were divided into three groups according to steroid treatment regimen after cochlear implantation. The first group of 18 patients (7 women and 11 men) were treated with intravenous steroid therapy (standard steroid therapy) (Figure 2); they were aged 32–73 years old (61.8 ± 11.9). The second group of 6 patients (4 women and 2 men) were treated with combined oral and intravenous steroid therapy (Figure 3); they were aged 53–73 (63.0 ± 6.7). The control group consisted of 5 patients (1 woman and 4 men) implanted without steroid therapy; they were aged 64–86 (73.2 ± 7.8). 

Two different medical products which were commercially prepared as two different formulations (oral tablets for oral administration and solution for injection) were administered to patients. Dexamethasone sodium phosphate was administrated intravenously for patients in the first and second subgroups. Prednisone was administrated orally only in the second subgroup in combination with dexamethasone sodium phosphate. Dexamethasone under the brand name Dexaven^®®^ (solution for injection, concentration of 4 mg/mL, 10 ampoules per package) and prednisone under the brand name Encorton^®®^ (tablets, 1, 5, 10, and 20 mg per tablet) were administered according to the protocol of the study.

### 2.3. Audiological Assessment and Preservation of Hearing

Audiological evaluation and hearing preservation were performed with pure tone audiometry. Frequencies ranged from 0.125 to 8 kHz (octaves and half-octaves). Measurements were carried out in the same soundproof cabin using the same diagnostic audiometer. The audiometer limit for each frequency was: 0.125 kHZ-90 dB; 0.250 kHZ-100 dB; 0.500 kHz–4 kHz-120 dB; 6 kHz-110 dB and 8 kHz-100 dB. Pure tone audiometry was performed before surgery, during activation (1 month after surgery), and in the 12th month after activation. Due to the long duration of the study, only half the patients underwent postoperative speech audiometry and speech audiometry (best Q/N, 65 dB/10 SNR).

### 2.4. Measures

The primary outcome measure was average hearing threshold across in all 11 frequencies (0.125–8 kHz) using both octaves and half-octaves according to the International Organization for Standardization ISO 8253-1:2010. All measurements were conducted in the same soundproof cabin by an experienced technician, using the same diagnostic audiometer, the Madsen Itera II (GN Otometrics, Hørskætten, Denmark) with calibrated earphones (TDH-39P) (Telephonics, Farmingdale, NY, USA). The secondary outcome measure was hearing preservation (HP) calculated by comparing hearing thresholds in the 1-year post-operative period with the preoperative hearing thresholds, according to the HP formula (Equation (1)) [21].

(1) $$\mathrm{HP}=\left(1-\frac{PTApost-PTApre}{PTAmax-PTApre}\right)*100\left[\%\right]$$

In this equation, PTA is pure tone average, measured at different time periods. PTA_PRE_ is the pure tone average measured in the preoperative period; PTA_POST_ is the pure tone average measured postoperatively; and PTA_MAX_ is the maximum sound intensity generated by a standard audiometer (usually 120 dB hearing level, HL). According to the HP classification, the results were divided into three groups: minimal hearing preservation (minimal HP) 0–25%; partial hearing preservation (partial HP) 26–75%; and complete hearing preservation (complete HP) > 75%.

### 2.5. Statistical Analysis

The assumption of normality was checked by analyzing the variables using the Kolgorov–Smirnov test. This assumption was completely violated in the intravenous group, partially violated in the oral and intravenous group, and was met only in the control group. For this reason, and because of small and unequal sample sizes, the non-parametric test was used in the statistical analysis. The Wilcoxon signed-rank test (T stands for test statistic) was used for testing differences between hearing thresholds in the preoperative period and in the 12-month post-activation period. A Kruskal–Wallis test (H stands for a test statistic) was performed to compare hearing thresholds from the three groups in each period separately. A chi-square test was used to assess the differences between the three groups in terms of hearing preservation. The required sample size was calculated with power 0.80 and alpha level 0.05. Results obtained in our previous study (2018, Skarzynska et al.) were used as an assumption. The sample size needed was established as n = 11 for each group. The analysis was blinded. The alpha-level was set to 0.05. All statistical tests were performed using IBM SPSS Statistics v24.

### 2.6. Subjects

Characteristics of the patients in terms of age, sex, operated ear and hearing thresholds (average across all frequencies) in non-operated ear are shown in Table 1. The three groups did not differ significantly in these characteristics. The PICO criteria was classified and described in the Table 2.

## 3. Results

Table 3 shows the pure tone average (PTA) results for each group in separate periods: before cochlear implantation, during activation (1 month after cochlear implantation) and 12 months in post-activation, separately for each group. It can be seen that at the activation period (1 month after surgery), the hearing thresholds started to deteriorate in each group. Averaged hearing thresholds for all patients, according to treatment type are shown in Table 3. 

Additionally, hearing thresholds were observed in the patients’ non-operated ears. They remained stable and were as follows:

(1) The intravenous group: the pre-op range was 32.86–110.45 db HL; M = 78.75 (SD = 30.30). The 12 months range was 31.36–110.45 db HL; M = 78.39 (SD = 28.54). The difference was not statistically significant: T = 0.05; *p* = 0.959.
(2) The oral and intravenous group: the pre-op range was 59.29–107.00 db HL; M = 76.70 (SD = 16.97). The 12 months range was 65.45–100.91 db HL; M = 77.80 (SD = 12.75). The difference was not statistically significant: T = 0.73; *p* = 0.463.
(3) The control group: the pre-op range was 26.25–89.09 db HL; M = 68.72 (SD = 25.59). The 12 months range was 24.09–91.36 db HL; M = 69.00 (SD = 27.13). Again, the difference was not statistically significant: T = 0.67; *p* = 0.500.

### 3.1. Within-Group Comparison of Hearing Thresholds

Average hearing thresholds were compared in the preoperative period and 12 months after activation for each group separately. The intravenous group had better hearing thresholds in the preoperative period than after 12 months, and the difference was statistically significant, T = 2.69; *p* = 0.007. The oral + intravenous group also had significantly better hearing thresholds in the preoperative period than after 12 months (*T* = 1.99; *p* = 0.046). The same was observed in the control group (*T* = 2.02; *p* = 0.043). In summary, deterioration of hearing thresholds was observed in all three groups. 

### 3.2. Between Group Comparison of Hearing Thresholds

Statistical analysis revealed that there were statistically significant differences between groups in the preoperative period (*H* = 8.87; *p* = 0.012); before surgery, the oral and intravenous group had slightly better hearing thresholds than either of the other two groups. However, these between-group differences disappeared 12 months after CI activation (*H* = 5.64; *p* = 0.060), when all three groups performed similarly. 

**Figure 4:**
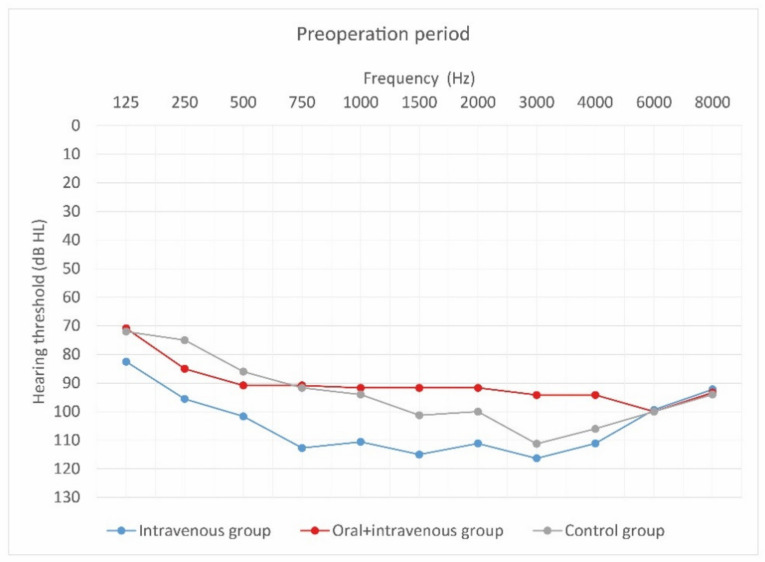
Mean hearing thresholds of patients with standard steroid therapy (i.v. group), patients with prolonged steroid therapy (oral + i.v. group) and control patients in the pre-operative period.

**Figure 5:**
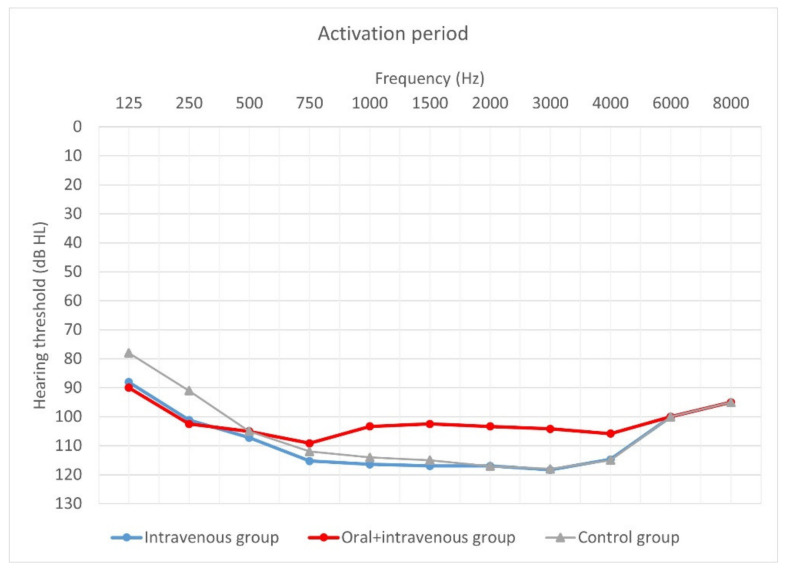
Mean hearing thresholds of patients with standard steroid therapy (i.v. group), patients with prolonged steroid therapy (oral + i.v. group) and control patients upon activation.

**Figure 6:**
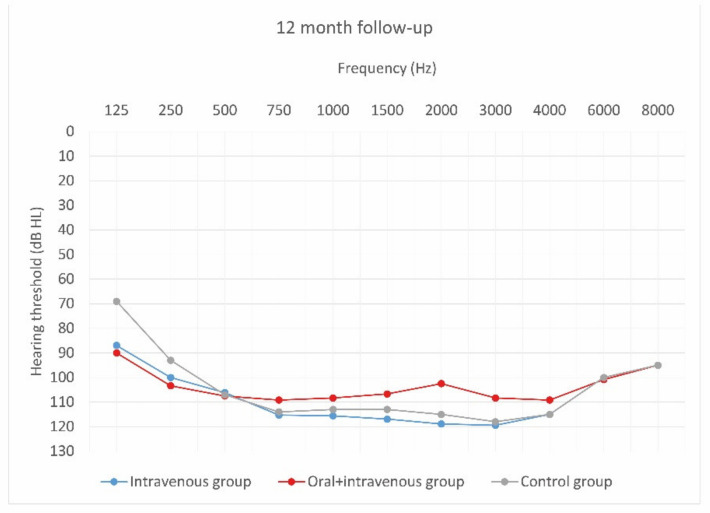
Mean hearing thresholds of patients with standard steroid therapy (i.v. group), patients with prolonged steroid therapy (oral + i.v. group) and control patients in the twelve-month follow-up after CI activation.

**Figure 7:**
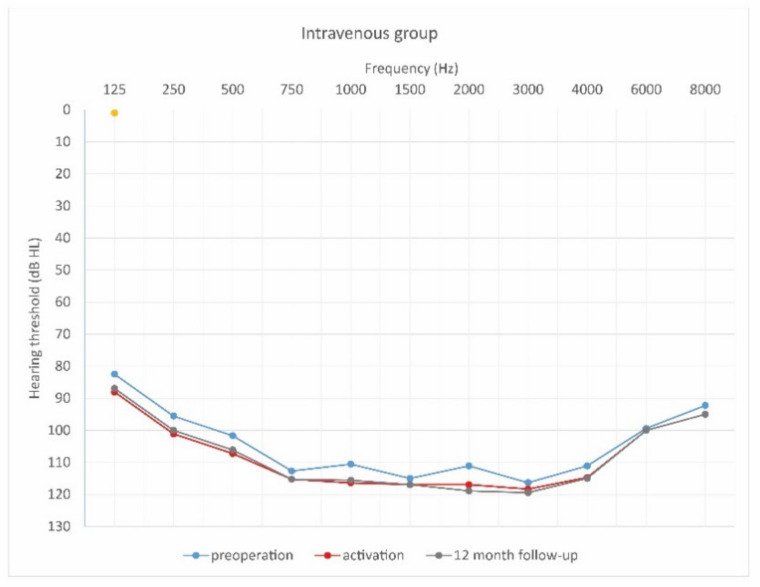
Mean hearing thresholds of patients with standard steroid therapy (i.v. group) in the pre-operative period, upon activation, and in the twelve-month follow-up after CI activation.

**Figure 8:**
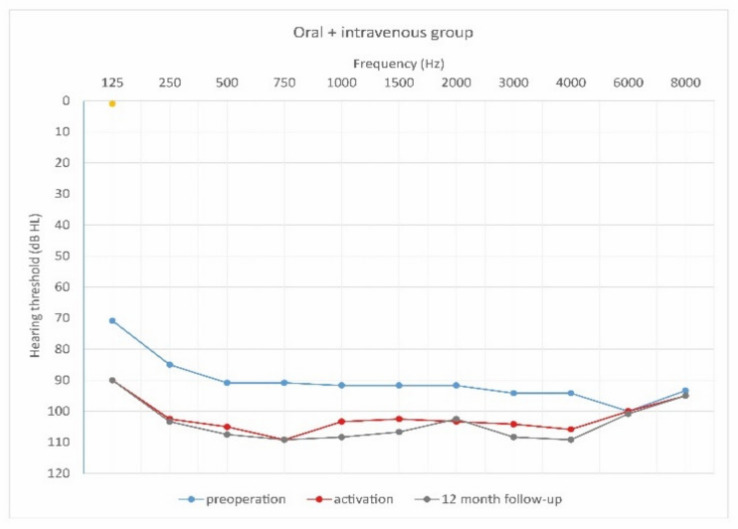
Mean hearing thresholds of patients with prolonged steroid therapy (oral and i.v. group) in the pre-operative period, upon activation, and in the twelve-month follow-up after CI activation.

**Figure 9:**
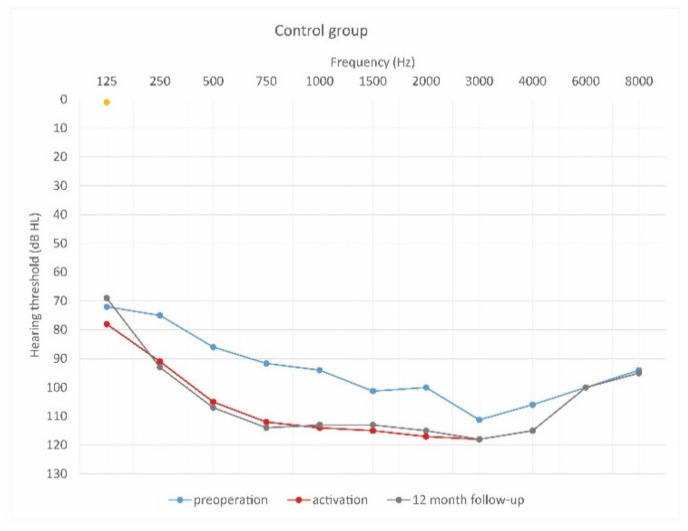
Mean hearing thresholds of control patients in the pre-operative period, upon activation, and in the twelve-month follow-up after CI activation.

Figure 4, Figure 5 and Figure 6 show hearing thresholds across all frequencies from 0.125 to 8 kHz in the preoperative period, at activation, and at 12-month follow-up for all three groups. Figure 7, Figure 8 and Figure 9 show hearing thresholds across frequencies from 0.125 to 8 kHz in each group separately. 

As can be seen, deterioration of hearing thresholds was observed in the patients regardless of the type of treatment.

### 3.3. Hearing Preservation

Data concerning hearing preservation converted to categories (no measurable hearing, minimal, partial, complete) are shown in Table 4. Hearing preservation rate was calculated according to the hearing preservation formula.

There was a statistically significant difference between the three groups in hearing preservation: χ^2^ = 13.74; *p* = 0.033. The majority of the patients with standard steroid therapy (72.2%) had no measurable hearing at 12 months after CI activation, while the results obtained by the patients in the oral and intravenous group were slightly better. The controls also had slightly better HP than patients in the intravenous group.

## 4. Discussion

Hearing preservation plays a vital role in cochlear implantation. The evidence shows that it is possible to preserve residual hearing (especially at low frequencies) by a combination of appropriate electric stimulation and steroid therapy, by which means hearing and speech in noise discrimination can be significantly improved [15]. Although commercial and research activity continues to develop better atraumatic implant electrodes, as well as techniques for atraumatic insertion of the electrode into the cochlea, clinically there is still a need for better pharmacological treatments to protect hair cells [15]. The role of steroids in hearing preservation has been examined clinically using different schemes and different animal models. Studies have demonstrated that prolonged administration of the steroid from an electrode previously coated with the drug, can significantly improve hearing preservation. 

A study from 2013 demonstrated that an electrode in which dexamethasone was incorporated could continuously release steroid into the inner ear [22]. Similar results were confirmed in 2012, when steroid was intra-tympanically applied in the preoperative period. Hearing preservation was significantly better (and had higher stability) in the intervention group than in the control group. The study showed that additional use of glucocorticoids intra-tympanically improved and stabilized hearing preservation for both adults and children with residual hearing [15]. The clinical effect of steroid on residual hearing was investigated in a gerbil animal model, when animals were implanted with an electrode coated with two different concentrations of dexamethasone (1% and 10%) in one ear and with a conventional electrode in the opposite ear [23,24,25]. Hearing levels were established based on tone burst ABR (auditory brainstem responses) at two different post implantation periods (4–6 weeks and 13 months), and the results showed significantly improved hearing at high frequencies (although not clear-cut at low frequencies [26]. 

As far as administration of steroid during cochlear implantation is concerned, Cho et al. compared the efficacy of steroid administration for protecting residual hearing during two separate periods (preoperatively and intraoperatively). Dexamethasone at a dose of 5 mg/mL was administered systemically before cochlear implantation and locally (topically) during surgery. The study protocol did not look at prolonged steroid administration, but there was a statistically significant difference between the steroid group and the control group. This conclusion supports the benefits of steroid pharmacotherapy, even 1 year after surgery [27]. 

However, different results came from a preliminary study published in 2018. Although the regimen, dose, and duration of administration of glucocorticoids in both studies was identical, the results of pure tone audiometry (PTA) were different. However, different cochlear electrodes and implants were used, and the number of patients enrolled in the study was also different (36 compared to 29). In addition, their ages were different (43–52, while in this study the age range was 61–73). All patients underwent follow-up evaluation. Note that it is not always possible to preserve residual hearing after implantation in patients of more advanced age. Hearing loss isolates the elderly from society, and contributes to depression and a decline in the quality of life. Cochlear implantation has been demonstrated to be a method that can preserve hearing in this group of patients. Unfortunately, there are some factors specific to the elderly that may affect the course of surgery, performance of an implant, and its long-term benefits. In general, these are degenerative changes in the auditory pathway, progressive central auditory dysfunction, cognitive and adaptive difficulties, long-term deafness, and comorbidities. Another important factor is surgical access to the cochlea. Often there is ossification at the beginning of the basal turn, meaning a surgical maneuver is needed in order to place the electrode properly. This requires additional drilling using a diamond burr of 0.8 or 1.0 mm and a speed of no more than 4000 rpm. All these factors can influence the perioperative period and cause complications [28,29].

The results of pure tone audiometry (PTA) were different. In the 2018 manuscript, the PTA frequency range was 0.125–8 kHz, as here, but PTA was assessed in four periods: preimplantation, during processor activation, 1 month after the activation, and 6 months after activation. Hearing preservation (HP) was calculated for the 6-month period. Here, PTA was estimated at three different times: pre-implantation, during activation (1 month after surgery), and 12 months after activation. HP was calculated over 12 months. The outcomes from both studies differ. In the 2018 study, hearing remained stable in the second subgroup (combined oral and IV steroid) during the follow-up period. Based on HP, they also had the highest overall values and the smallest variability. The results of the study showed that steroid therapy stabilizes hearing thresholds and preserves hearing in adults, and that a combination of i.v. and oral steroid therapy is an optimal treatment regimen. After 12 months there was a clear advantage of a combined steroid regimen. The majority of patients had complete (80%) or partial (20%) hearing preservation [14]. 

Our results with the Oticon implant are different. Deterioration of hearing thresholds was observed regardless of the type of treatment. The majority of patients with standard steroid therapy (72%) had no measurable hearing 12 months after activation. The patients from both the other groups (the second and the control group) had slightly better hearing preservation and the difference between groups was statistically significant. Average hearing thresholds were compared in the preoperative period and 12 months after surgery. The intravenous group (the first subgroup) had better hearing thresholds in the preoperative period than after 12 months, with the difference being statistically significant. The same was observed in the control group. To sum up, a deterioration of hearing thresholds was observed in all three groups. Statistical analysis revealed that there were statistically significant differences between groups in the preoperative period. Before surgery, the oral and intravenous group had slightly better hearing thresholds than either of the other two groups. 

The Oticon Neuro Zti EVO implant became available in 2015 and from 2017 this type of implant was included in this project. The clinical experience with the Oticon implant is therefore limited, and research on the use of corticoids along with it is still at the pioneering stage. On the other hand, the Med-El implant was launched many years earlier, although only introduced into this study from 2018. Additionally, due to the positive results for patients obtained as early as 2018, the decision to continue the studies with the same regimes of corticosteroid administration—but with a different cochlear implant—was accepted by researchers (and by our Ethics Committee) as a feasible, safe, and important project. However, the inclusion criteria for the present project were slightly different from those used in the 2018 study due to different inclusion criteria for implantation with the Oticon Neuro Zti EVO.

The decision to administer corticosteroids orally and/or intravenously is important from the perspective of patients and surgeons. This study adds to our knowledge of pharmacotherapy as a part of surgical technique. It is the first prospective study involving the Oticon Neuro Zti implant which compares different corticoid regimens before, during, and after surgery. From a pharmacokinetic point of view the most direct form of corticoid administration is intracochlear, but this route is the most challenging. At the present time, there is no authorization by the EMA (European Medical Agency) or the FDA (Food and Drug Administration) for any drug to be delivered intracochlearly. Any drug delivered this way is administered off-label. The administration of corticoids via the routes used here accords with our Ethics Committee decision about the characteristics of this medical product. According to the inclusion criteria of hearing thresholds approved by the bioethical committee, the low performance before surgery may have affected the final result that there is no hearing preservation in the patients. One reason may be the durations of the corticoids delivery. The dose level of dexamethasone and prednisone is to be optimal according the Summary of the Product Characteristics, and the ratio between risk of adverse effects and benefits of the treatment has been well rated.

### Limitations

The limitation of this study is the number of patients included. We planned to have at least 11 patients per group, so initially we recruited more than 40 patients. However, some dropped out of the study, and we also have patients who were recruited a few months ago and have not yet completed the 12-month follow-up yet. This work is part of an ongoing project that aims to assess the effectiveness of steroids in protecting residual hearing in different groups of patients and with different cochlear implant systems.

## 5. Conclusions

To the best of our knowledge, this study is the first prospective clinical study to compare the results of different ways of administering glucocorticoids (dexamethasone and prednisone) in patients who had undergone cochlear implantation with the Oticon Medical Neuro cochlear implant system (Neuro Zti Implant, the Neuro 2 sound processor, and Neuro Zti EVO atraumatic electrode carrier). There are a number of factors that affect the results, effectiveness, and final patient satisfaction after cochlear implantation. One important factor is the age of the patient. The results of this study also demonstrate that the type of cochlear implant, and its electrode properties (such as flexibility) play a key role in stabilizing hearing thresholds and preserving residual hearing. The outcomes of the study relate not only to the type of electrode, but also to the age of the patients, which in our study was relatively advanced. It is not always possible to save residual hearing in elderly patients, even when additional pharmacotherapy with corticoids is administered. In such cases, the result of administration of steroids is a minor consideration, as is the type of implant. Therefore, in this study we found that, even though they underwent implantation with the Oticon Zti EVO, the steroid regimen itself did not play a significant role in preserving the hearing of these patients, who had non-functional residual hearing.

## Figures and Tables

**Table 1:** Characteristics of patients.

	Intravenous (IV) Group (n = 18)	Oral and IV Group (n = 6)	Control Group (n = 5)	Test Result
Age (years)	32–73; 61.8 ± 11.9	53–73; 63.0 ± 6.7	64–85; 73.2 ± 7.8	*H* = 5.45; *p* = 0.065
Sex	11 M; 7 F	2 M; 4 F	4 M; 1 F	*χ*^2^ = 2.57; *p* = 0.277
Operated ear	8 R; 10 L	3 R; 3 L	3 R; 2 L	*χ*^2^ = 0.39; *p* = 0.824
Hearing thresholds in the non-operated ear	32.9–110.5; 78.8 ± 30.3	59.3–107.0; 76.7 ± 20.0	26.3–89.1; 68.7 ± 25.6	*H* = 0.53; *p* = 0.767

Age and hearing thresholds expressed as range and mean ± standard deviation. M, male; F, female; R, right ear; L, left ear; H, the result of the Kruskal-Wallis test; χ^2^, the result of the chi square test; *p*—significance level.

**Table 2:** Shows the PICO classification (P—population, I—intervention, C—comparison, O—outcome) in which each component is precisely described.

Population (P)	≥18 years of age with: Hearing levels in the range of 65–120 dB at 0.25–1 kHz; Hearing levels of 75–120 dB at 2–8 kHz
Intervention (I)	Administration of steroids in two different regimens: Dexamethasone was administrated intravenously (0.1 mg per kg of body mass) 30 min before the cochlear implant surgery. The same dose was administered every 12 h for 3 consecutive days (6 doses). The dexamethasone used in this study was supplied in ampoules of 2 mL solution (4 mg/mL) Or Prednisone was administrated orally at a dose of 1 mg per kg of body mass 3 days prior to surgery. Then 30 min before implantation surgery, dexamethasone at a dose of 0.1 mg per kg body mass was administered IV (as with the first group). Over the next 3 days, prednisone was administrated orally (1 mg of prednisone per kg body mass). After this time, the dose was reduced by about 10 mg per day until it reached zero. To investigate the effects of prolonged steroid administration, we chose to compare the IV and oral administration routes
Comparison (C)	No administration of steroid
Outcome (O)	The desired outcomes were the smallest deterioration in the average pure tone audiometry results after 12 months in comparison with preoperative period, with the assumption that the results after surgery will be higher than before surgery

**Table 3:** Hearing thresholds of patients at each of the three study periods.

Group		Min	Max	M	SD	Me
Intravenous	Pre	67.14	110.45	102.85	10.94	107.73
	1 m	94.09	110.45	108.18	4.52	110.45
	12 m	87.73	110.45	108.11	5.63	110.45
Oral and intravenous	Pre	75.71	103.89	89.40	10.49	91.88
	1 m	90.00	109.09	101.89	6.99	102.05
	12 m	90.00	110.45	103.71	7.47	103.86
Controls	Pre	80.45	102.00	92.97	10.11	97.73
	1 m	96.82	110.45	105.45	5.64	106.82
	12 m	101.36	110.45	104.73	3.47	104.55

Min, minimum; Max, maximum; M, mean; SD, standard deviation; Me, median.

**Table 4:** Hearing preservation 12 months after CI activation, according to treatment regime.

	No Measurable Hearing	Minimal	Partial	Complete
Intravenous group (IV)	13 (72.2)	0 (0.0)	3 (16.7)	2 (11.1)
Oral + IV group	2 (33.3)	2 (33.3)	1 (16.7)	1 (16.7)
Control group	1 (20.0)	0 (0.0)	3 (60.0)	1 (20.0)

Data are given as the number of patients (with percentage in brackets).
